# Heparin Promotes Cardiac Differentiation of Human Pluripotent Stem Cells in Chemically Defined Albumin‐Free Medium, Enabling Consistent Manufacture of Cardiomyocytes

**DOI:** 10.5966/sctm.2015-0428

**Published:** 2016-09-02

**Authors:** Yongshun Lin, Kaari L. Linask, Barbara Mallon, Kory Johnson, Michael Klein, Jeanette Beers, Wen Xie, Yubin Du, Chengyu Liu, Yinzhi Lai, Jizhong Zou, Mark Haigney, Hushan Yang, Mahendra Rao, Guokai Chen

**Affiliations:** ^1^ National Heart, Lung and Blood Institute, NIH, Bethesda, Maryland, USA; ^2^ NIH Stem Cell Unit, NIH, Bethesda, Maryland, USA; ^3^ National Institute of Neurological Disorders and Stroke, NIH, Bethesda, Maryland, USA; ^4^ Uniformed Services University of Health Sciences, Bethesda, Maryland, USA; ^5^ Thomas Jefferson University, Philadelphia, Pennsylvania, USA; ^6^ Center for Regenerative Medicine, NIH, Bethesda, Maryland, USA; ^7^ Faculty of Health Sciences, University of Macau, Tapai, Macau, People's Republic of China

**Keywords:** Cardiac, Cell culture, Embryonic stem cells, Induced pluripotent stem cells, Heparin

## Abstract

Cardiomyocytes can be differentiated from human pluripotent stem cells (hPSCs) in defined conditions, but efficient and consistent cardiomyocyte differentiation often requires expensive reagents such as B27 supplement or recombinant albumin. Using a chemically defined albumin‐free (E8 basal) medium, we identified heparin as a novel factor that significantly promotes cardiomyocyte differentiation efficiency, and developed an efficient method to differentiate hPSCs into cardiomyocytes. The treatment with heparin helped cardiomyocyte differentiation consistently reach at least 80% purity (up to 95%) from more than 10 different hPSC lines in chemically defined Dulbecco's modified Eagle's medium/F‐12‐based medium on either Matrigel or defined matrices like vitronectin and Synthemax. One of heparin's main functions was to act as a Wnt modulator that helped promote robust and consistent cardiomyocyte production. Our study provides an efficient, reliable, and cost‐effective method for cardiomyocyte derivation from hPSCs that can be used for potential large‐scale drug screening, disease modeling, and future cellular therapies. Stem Cells Translational Medicine *2017;6:527–538*

**Significance Statement**

Using a new method, heparin was identified as a novel reagent that promotes cardiomyocyte differentiation. Albumin was not necessary for cardiac differentiation when heparin was present. Heparin has biphasic regulations of Wnt signaling during cardiac differentiation. This study reports that the heparin‐based cardiac differentiation method is easier in terms of quality control and is cost‐effective for large‐scale production and potential clinical applications.

## Introduction

Human pluripotent stem cells (hPSCs), including embryonic stem cells (ESCs) and induced pluripotent stem cells (iPSCs), hold great promise in drug discovery and future cellular therapy 1, 2, 3, 4. Cardiomyocytes derived from hPSCs have been in great demand because of their applications in drug screening and evaluation of cardiac cytotoxicity, as well as their potential for cellular replacement therapy 5, 6. To generate large numbers of cardiomyocytes for these efforts, multiple serum‐containing or serum‐free differentiation conditions have been reported over the past decade 5, 7, 8, 9, 10, 11, 12. Despite different growth factor and inhibitor combinations used in various serum‐free procedures, most conditions contain albumin or albumin‐containing supplements, such as B27, which either contain animal products (e.g., bovine serum albumin) or serum substitutes that introduce variability due to batch‐to‐batch inconsistencies of the source materials. Albumin‐free conditions were recently reported with careful modulations of Wnt pathway activities during differentiation 13, but it is still desirable to seek novel solutions to improve the efficiency and consistency of cardiomyocyte differentiation in albumin‐free conditions. We previously developed an albumin‐free E8 medium for hPSC derivation and maintenance 14. E8‐based media are suitable for screening novel albumin‐free conditions for hPSC cardiomyocyte differentiation.

Albumin, a major serum component, has long been used in somatic and stem cell culture and plays an important role in the cells. Albumin is not only a carrier for lipids and other bioactive factors, such as vitamins and fatty acids, but is also involved in biophysical modulation as a protective reagent and colloid osmolality regulator 15. The complicated roles of albumin in cell culture often prevent the discovery of other important regulatory factors in stem cell culture. Dulbecco's modified Eagle's medium (DMEM)/F‐12‐based E8 medium is an albumin‐free stem cell medium that serves as a good starting point to develop albumin‐free differentiation conditions for cardiomyocytes, but there have been no novel protocols reported using E8 medium 8, 11. In this study, we sought to identify novel conditions and develop an efficient method for hPSC cardiac differentiation using the albumin‐free E8 medium platform.

Here, we report heparin as a novel factor promoting cardiomyocyte differentiation in the absence of albumin. Signaling pathway analysis indicated that heparin modulated Wnt to promote cardiomyocyte differentiation. We optimized a robust cardiomyocyte differentiation protocol that has been reproducibly applied on more than 10 hESCs and human induced PSC lines. This is the most cost‐effective protocol to date, to our knowledge, that produces an efficiency and consistency of cardiac differentiation comparable to that of previously reported protocols. Applying this protocol, we could provide large‐scale production of high‐quality hPSC‐derived cardiomyocytes at low cost for drug screening and future cellular therapies.

## Materials and Methods

### hPSC Maintenance

Human ESCs (H1 and H9) and iPSCs were maintained in E8 media (Essential 8 Medium; Thermo Fisher Scientific Life Sciences, Waltham, MA, http://www.thermofisher.com) on Matrigel‐coated (10 μg/cm^2^) plates with daily medium changes. Cells were passaged with EDTA, as described previously 16. Briefly, 60%–70% confluent hPSCs were washed with 0.5 mM EDTA/phosphate‐buffered saline (PBS) once and incubated in 0.5 mM EDTA/PBS for 3–5 minutes at room temperature. EDTA/PBS was removed from the plate and the cells were gently washed off via E8 medium containing Rho‐associated protein kinase (Rock) inhibitor and passaged onto Matrigel‐coated plates at 1:6 to 1:8 dilutions. Rock inhibitor was removed on the next day after passage.

### Cardiomyocyte Differentiation in Monolayer

When hPSCs had grown to 80%–90% confluence 2–3 days after plating, the medium was changed from E8 to differentiation basal medium, which contains E8 basal medium (DMEM/F‐12, l‐ascorbic acid, selenium, transferrin, and NaHCO_3_) 14, 1× Chemically Defined Lipid Concentrate (100×, catalog no. 11905‐031; Thermo Fisher Scientific Life Sciences) and 1× penicillin‐streptomycin (catalog no. 15140‐122; Thermo Fisher Scientific Life Sciences). The day was defined as day 0. CHIR99021 (5 μM, catalog no. 4423; Tocris Bioscience, Avonmouth, Bristol, UK, https://www.tocris.com) was added to the differentiation basal medium at day 0 for 24 hours. IWP2 (3 μM, catalog no. 3533; Tocris Bioscience) was added from day 2 to day 5. Heparin was added to the medium at the indicated dosages and times. Insulin (20 μg/ml, catalog no. I9278; Sigma‐Aldrich, St. Louis, MO, http://www.sigmaaldrich.com) was added to differentiation basal medium to maintain cardiomyocytes from day 7 onward. The medium was changed daily until day 7, but changed every 2–3 days from day 7 onward. The growth factors, inhibitors, and enzymes used in culture were as follows: activin A (10 ng/ml, catalog no. 338‐AC/CF; R&D Systems, Minneapolis, MN, https://www.rndsystems.com), BMP4 (10 ng/ml, catalog no. 314‐BP/CF; R&D Systems), fibroblast growth factor 2 (FGF2; 100 ng/ml, catalog no. 100‐18B; Peprotech, Rocky Hill, NJ, https://www.peprotech.com), transforming growth factor β (TGFβ) 1 (1.74 ng/ml, catalog no. 240‐B/CF; R&D), dorsomorphin (3 μM, catalog no. 04‐0024; Stemgent, Lexington, MA, https://www.stemgent.com), LDN‐193189 (0.1 μM, catalog no. 04‐0074; Stemgent), PD0325901 (1 μM, catalog no. 04‐0008; Stemgent), SB431542 (3 μM, catalog no. s1067; Selleckchem, Houston, TX, http://www.selleckchem.com), heparin (catalog no. H3149; Sigma‐Aldrich), heparinase I (catalog no. P0735S; New England Biolabs, Ipswich, MA, https://www.neb.com).

### Real‐Time Polymerase Chain Reaction

RNA was purified using TRI Reagent Solution according to the manufacturer's protocol (Thermo Fisher Scientific Life Sciences). Residual DNA was removed using the TURBO DNA‐free kit. Reverse transcription was carried out with Maxima H Minus Reverse Transcriptase (Thermo Fisher Scientific Life Sciences) primed with Poly N15‐mer (Eurofins, Luxembourg, http://www.eurofins.com) with the recommended protocol. Before polymerase chain reaction (PCR), the RNA template was removed with addition of Ambion Ribonuclease H (Thermo Fisher Scientific Life Sciences) from *Escherichia coli*. Real‐time PCR was performed on the BIO‐RAD CFX96 (Bio‐Rad Laboratories, Hercules, CA, http://www.bio‐rad.com) using SsoAdvanced Universal SYBR Green Supermix (Bio‐Rad Laboratories). PCR primers are listed in supplemental online Table 1.

### Immunofluorescence Staining

Cells cultured in monolayer were fixed in 2% paraformaldehyde (PFA) for 10 minutes at room temperature, washed with PBS, and permeabilized with 0.2% Triton X‐100/PBS for 3 minutes. After successive washes in PBS, the cells were blocked in 10 mg/ml bovine serum albumin (BSA) for 30 minutes and incubated with primary antibody for 1 hour at room temperature. Cells were then washed 3 times with PBS for 5 minutes and incubated with secondary antibodies (Alexa 488 or 594‐conjugated; Thermo Fisher Scientific Life Sciences) for 1 hour. Then 4′,6‐diamidino‐2‐phenylindole (DAPI) was applied for nuclei staining. The following primary antibodies were used for immunofluorescence: α‐actinin (mouse, 1:1,000, catalog no. A7811; Sigma‐Aldrich), cardiac troponin I (CTNI; rabbit, 1:200, catalog no. sc‐15368; Santa Cruz Biotechnologies, Dallas, TX, http://www.scbt.com), cardiac troponin T (CTNT; mouse, CT3, 1:1,000; Developmental Studies Hybridoma Bank, Iowa City, IA, http://dshb.biology.uiowa.edu), desmin (mouse, 1:100, catalog no. M0760; Agilent Technologies, Glostrup, Denmark, http://www.dako.com) NKX2.5 (rabbit, 1:200, catalog no. ab35842; Abcam, Cambridge, MA, http://www.abcam.com), MLC2a (MYL7, mouse, 1:100, catalog no. 311011; Synaptic Systems, Goettingen, Germany, https://www.sysy.com), and MLC2v (MYL2, rabbit, 1:100, catalog no. 10906‐1‐AP; ProteinTech, Chicago, IL, http://www.ptglab.com)

### Flow Cytometry Assay

Cells differentiated for 10 days in monolayer were dissociated with TrypLE (Thermo Fisher Scientific Life Sciences) for 5 minutes. Single cells were washed off and suspended by DMEM/10% fetal bovine serum, followed by centrifugation. The pellets were washed with PBS once and fixed by 2% PFA for 5 minutes. After washing with PBS and centrifuging, permeabilization was performed by 0.3% Triton X‐100 for 3 minutes. The cells were centrifuged and resuspended with 1% BSA to block unspecific binding of antibody, followed by incubation with primary antibodies (diluted in 1% BSA) for 1 hour. After washing, the cells were incubated with second antibodies conjugated with fluorescein isothiocyanate or phycoerythrin (1:1,000 diluted in PBS; Thermo Fisher Scientific Life Sciences) for 30 minutes. The cells were washed once and suspended in PBS for the flow cytometry assay.

### Electrophysiological Assay

Cardiomyocytes were dissociated and plated on glass coverslips in culture medium before use. For action potential measurements, cells were placed in a small‐volume chamber and bathed in Tyrode solution containing 140 mM NaCl, 5.4 mM KCl, 1.8 mM CaCl_2_, 1 mM MgCl_2_, 10 mM HEPES, and 10 mM glucose, at a pH of 7.4 and maintained at 33°C–35°C. Action potentials were recorded using a patch clamp amplifier (Axopatch 200B; Molecular Devices, Sunnyvale, CA, https://www.moleculardevices.com) in whole‐cell current‐clamp mode. Pipettes (1–3 MOhm) were filled with internal solution containing 100 mM K‐aspartate, 30 mM KCl, 10 mM HEPES, 1 mM MgCl_2_, 5 mM Mg‐ATP, 5 mM Na_2_ creatine phosphate, 0.1 mM EGTA, and mM 0.025 CaCl_2_ (50 nM free Ca^2+^), at a pH of 7.2.

### Transmission Electron Microscopy

Cells were fixed in 2.5% glutaraldehyde, 0.1 M sodium cacodylate buffer, at a pH of 7.4 for 90 minutes at room temperature. Cells were postfixed with osmium tetroxide, stained en bloc with uranyl acetate, dehydrated in an ethanol series, and embedded in epoxy resin. Chemicals were from Electron Microscopy Sciences (Hatfield, PA, https://www.emsdiasum.com). Thin sections were cut parallel to the adherent surface, poststained with uranyl acetate and lead citrate, and viewed with a JEM‐1200EX electron microscope (JEOL USA, Peabody, MA, http://www.jeolusa.com) equipped with an AMT XR‐60 digital camera (Advanced Microscopy Techniques, Woburn, MA, http://www.amtimaging.com).

#### Microarray Analysis

Global gene expression data were generated per sample using Agilent's One Color Gene Expression Oligo arrays in accordance with the manufacturer's guidelines (Agilent Technologies, Glostrup, Denmark, http://www.agilent.com). Statistical analysis of the data was performed in R (http://cran.r‐project.org/). Raw expression data generated across samples were first pedestaled by 2, log2 transformed, then quantile normalized. Then the quality of the data was assured via sample‐level Tukey box plot, covariance‐based principal components analysis scatter plot, and correlation‐based heatmap in R. Gene probes not having at least one expression measurement greater than system noise were deemed “noise biased” and discarded. System noise was defined as the lowest observed expression value at which the locally weighted scatter plot smoothing (LOWESS) fit of the data (CV is approximately the mean) grossly deviated from linearity. For gene probes not discarded, expression measurements were floored to equal system noise if they were less than system noise and the LOWESS fit itself used in conjunction with the random normal distribution to construct four virtual expression values per actual observed value. These values were then subject to one factor analysis of variance testing under the Benjamini and Hochberg false discovery rate multiple comparison correction condition using Time::Protocol as the factor.

Gene probes found to have a corrected *p*‐value ≥.05 by this test were discarded, and the remaining probes were subjected to the Tukey honestly significant difference post hoc test. Gene probes with a post hoc *p*‐value <.05 and a difference of means of ≥1.50 were subset as having expression “significantly different” between the Time::Protocol conditions being compared. Annotation of subset gene probes was accomplished using IPA (QIAGEN, Redwood City, CA, http://www.ingenuity.com). For gene probes annotated in IPA as cardiogenesis related, expression at days 6 and 10 was subset and compared via Pearson correlation with the average expression observed at days 6 and 10 for probes representing genes *TNNT2* and *NKX2‐5*. Gene probes found to have a significant (*p* < .05) positive correlation were summarized by heatmap and construed to be the cardiogenesis genes involved at days 6 and 10. IPA was also used to explore and compare gene expression differences ongoing in the Wnt signaling pathway at days 6 and 10. Differences in this pathway were also summarized by heatmap.

### Myocardial Infarction and Cardiomyocyte Injection

Myocardial infarction was performed by ligation of the left anterior descending coronary artery in NSG mice (Jackson Laboratory, Bar Harbor, ME, https://www.jax.org) under anesthesia of 1%–3% isoflurane. hPSCs‐derived cardiomyocytes (5 × 10^5^/20 μl) were injected into the myocardium at the border zone of the infarct area through a 29‐gauge needle. At 7 weeks after surgery, the animals were killed, and the hearts were removed and perfused with 4% paraformaldehyde for immunostaining. All mouse protocols were reviewed and approved by National Heart, Lung, and Blood Institute, U.S. National Institutes of Health, Animal Care and Use Committee.

### Statistical Analysis

All data are presented as the mean ± SD of three or more independent experiments. Significance was determined by Student's *t* test.

## Results

### Heparin Enhances hPSC Cardiomyocyte Differentiation Along With Wnt Modulation in E8‐Based Medium

**Figure 1:**
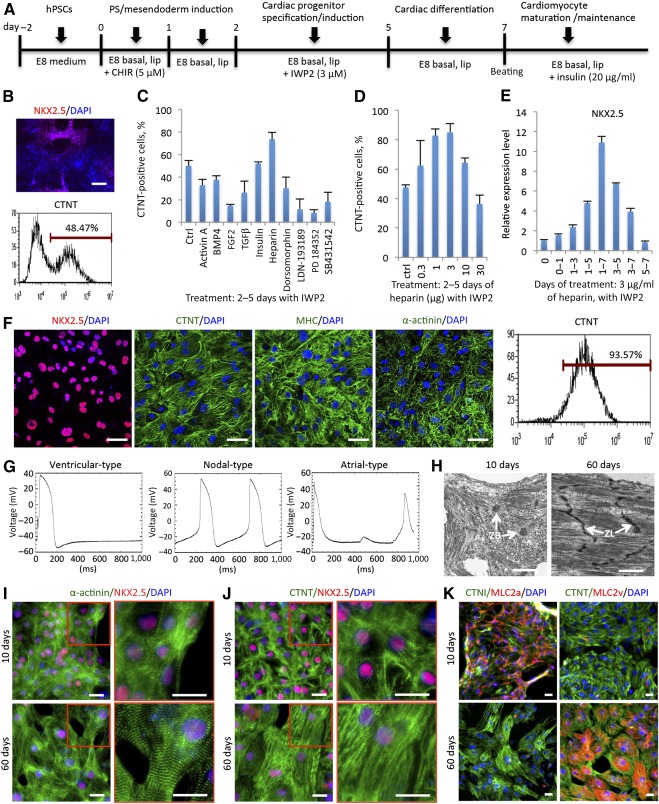
Heparin enhances hPSC cardiomyocyte differentiation along with Wnt modulation in E8 medium. **(A):** Schematic of hPSC differentiation protocol with singular modulation of Wnt signaling in the absence of B27. The experiments were carried out in chemically defined E8 basal medium (Dulbecco's modified Eagle's medium/F‐12 plus l‐ascorbic acid, selenium, and transferrin) with chemically defined lipid concentrate. **(B):** Cardiac differentiation efficiency without B27 in an E8‐based platform displayed by immunofluorescence of NKX2.5 (top) and flow cytometry of CTNT (bottom). Scale bar = 100 μm. **(C):** Screen of signaling pathway regulation during cardiac specification (treated at days 2–5). Data are presented as the mean ± SD of three or more independent experiments. **(D):** Heparin dosage screen for CTNT‐positive cell percentage by flow cytometry at day 10 when treated at days 2–5 in the presence of Wnt inhibitor IWP2. Data are presented as the mean ± SD of three or more independent experiments. **(E):** Real‐time polymerase chain reaction of NKX2.5 indicates the effect of various heparin time‐course (3 μg/ml) treatments in the presence of the Wnt inhibitor IWP2. Data are presented as the mean ± SD of three or more independent experiments. **(F):** Confocal immunofluorescence and flow cytometry of cardiac markers indicate that the derived cardiomyocyte population was up to 95% pure with heparin and IWP2 treatment. **(G):** Electrophysiological characterization by representative action potential activities of 3 cardiomyocyte subtypes at day 30 after differentiation (*n* = 3 cells for atrial and ventricular types and *n* = 2 cells for nodal type). **(H–K):** Maturation of cardiomyocytes on culture plates. Transmission electron microscopy showed the more highly organized and clearly aligned myofibrils of day‐60 cardiomyocytes compared with that of day‐10 cells, as well as the Z‐lines of myofibril bundles at day 60 rather than the Z‐bodies of premyofibrils at day 10 **(H)**. Immunofluorescence of α‐actinin **(I)** and CTNT **(J)** showed the increased sarcomere alignment in cardiomyocytes from 10 days to 60 days of culture. **(K):** Costaining of MLC2a and MLC2v with CTNI or CTNT showed that the majority of cells at early stage (10 days) expressed MLC2a, whereas the majority of cells at late stage (60 days) expressed MLC2v. **(H):** Scale bar = 1 μm. **(F, I–K):** Scale bar = 25 μm. Abbreviations: CTNI, cardiac troponin I; CTNT, cardiac troponin T; ctrl, control; hep, heparin; DAPI, 4′,6‐diamidino‐2‐phenylindole; hPSC, human pluripotent stem cell; lip, chemically defined lipid; MHC, myosin heavy chain; MLC2a, myosin light chain 2a; MLC2v, myosin light chain 2v; PS, primitive streak; ZB, Z‐body; ZL, Z‐line.

To develop an albumin‐free condition for cardiomyocyte differentiation, we first adapted the strategy of singular modulation of Wnt signaling that was reported in high efficiency when adding BSA‐containing B27 supplement 8 to the E8 medium platform. An E8 basal medium (E8 without NaHCO_3_, insulin, bFGF, and TGFβ) for differentiation was established for human H1 ESCs with initial Wnt activation (GSK3β inhibitor, CHIR99021; Tocris Biostechne, Abingdon, U.K., https://www.tocris.com) at day 0–1 followed by Wnt suppression by the Wnt secretion inhibitor IWP2 at days 2–5. Albumin and B27 were purposely dropped out of the whole process (Fig. 1A). Consistent with previous reports 8, 11, beating cardiomyocytes emerged at day 7 after differentiation without the addition of B27 supplement or human recombinant albumin, but rarely exceeded 50% purity at day 10, as shown by the expression of cardiac markers NKX2.5 and CTNT (Fig. 1B). This result indicates that additional regulation is necessary for more robust cardiomyocyte differentiation, and that the procedure shown in Figure 1A could be used as a screening platform to identify novel promoting regulators in the absence of albumin.

Based on current understanding of cardiac differentiation, multiple signaling pathways (e.g., TGFβ, BMP, FGF, activin/nodal) crosstalk with Wnt signaling in regulating mesoderm induction and cardiac differentiation 5, 17. We sought to test whether these growth factors or their specific inhibitors altered the Wnt‐modulated cardiac derivation at different stages in the E8‐based medium. As a proteoglycan, heparin was also added to the screen list because it plays an essential role in interacting with and regulating multiple growth factor signaling pathways 18, 19, 20, 21. As shown in Figure 1C, all growth factors and inhibitors were included at commonly used dosages. Modulation of conventional signaling pathways by growth factors (i.e., TGFβ, BMP, FGF, activin/nodal) and their specific inhibitors did not improve cardiomyocyte differentiation efficiency, which is consistent with the consensus that Wnt inhibition is the main player promoting cardiac differentiation after mesoderm induction. To our surprise, the treatment of heparin from day 2 to day 5 significantly enhanced the efficiency of cardiac differentiation (Fig. 1C). Heparin, a proteoglycan regulating multiple growth factors, likely did not function through FGF pathways for this effect, because neither FGF2 nor the FGF pathway inhibitor improved cardiomyocyte differentiation in the screen.

The impact of heparin on cardiac differentiation was not previously reported to our knowledge, so we further confirmed its effect by evaluating the impact with time‐course and dosage experiments. The heparin treatment from day 1 to day 7 at 1–10 μg/ml produced the highest yields of CTNT‐ and NKX2.5‐positive cardiac population. The percentage yield was greater than 80% without further purification or lactate treatment 11 (Fig. 1D, 1E; supplemental online Fig. 1; supplemental online Video 1). Beginning with the plating density of H1 ESCs at 4 × 10^4^ cells per cm^2^ at day −2, the total number of live cells (>80% were cardiomyocytes) derived after 10 days of differentiation was approximately 5 × 10^5^ to 10 × 10^5^ cells per cm^2^ (supplemental online Fig. 1B, 1C), which increased cell density more than 10‐fold. The derived cardiomyocytes were identified by positive confocal immunofluorescence of multiple cardiac markers, and flow cytometry results showed that the population of CTNT‐positive cells could reach as high as 95% (Fig. 1F). Applying patch‐clamp analysis, we observed the action potentials of all three cardiomyocyte subtypes (ventricular, nodal, and atrial) in the derived cardiomyocyte population with heparin/IWP2 treatment (Fig. 1G).

To investigate the maturation potential of the derived cardiomyocytes, we compared cells after 2 months of differentiation, with cells after 10 days of differentiation. The ultrastructure images from transmission electron microscopy (TEM) distinguished the misalignment and low density of myofibrils in day‐10 cardiomyocytes compared with the well‐aligned high‐density myofibrils in day‐60 cardiomyocytes (Fig. 1H). Meanwhile, the TEM images also showed the punctate aggregate Z‐bodies in day‐10 cardiomyocytes compared with linear Z‐bands in day‐60 cardiomyocytes (Fig. 1H). These both indicate the sarcomere maturation from premyofibrils into mature myofibrils 22 Consistently, immunofluorescence of α‐actinin and CTNT showed a significant increase in sarcomere alignment at day 60 when compared with day 10 (Fig. 1I, 1J), suggesting the maturation of these cardiomyocytes by day 60 23.

The switched expression of two major isoforms of myosin light chain 2, MLC2a and MLC2v, has also been applied to indicate the maturation of cardiomyocytes. In developing hearts and early culture stages, MLC2a is expressed in the majority of both atrial and ventricular cardiomyocytes. In adult hearts and long‐term culture stages, matured ventricular cardiomyocytes gradually lose MLC2a expression but gain expression of MLC2v 8, 11. We costained MLC2a and MLC2v with cardiac troponins on derived cardiomyocytes at 10 days and 60 days after differentiation, respectively (Fig. 1K). At day 10, the majority of cardiomyocytes (CTNI positive) expressed MLC2a but not MLC2v; whereas at day 60, the majority of cardiomyocytes (CTNT positive) expressed MLC2v but not MLC2a. All these data demonstrated the maturation of these cardiomyocytes after long‐term culture.

### Heparin Alone Promoted Cardiac Differentiation From hPSCs Without Treatment of Wnt Inhibitors

**Figure 2:**
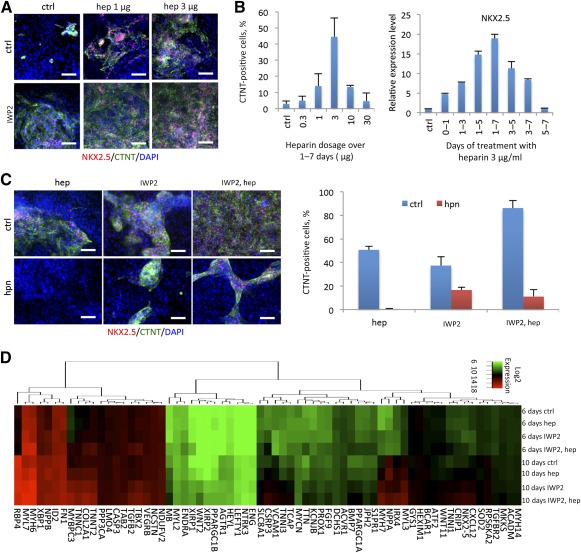
Heparin alone promoted cardiac differentiation from human pluripotent stem cells without treatment of Wnt inhibitors. **(A):** Immunofluorescence for NKX2.5 (red) and CTNT‐positive cells (green) at day 10 after treatment with 1 μg/ml or 3 μg/ml heparin in the absence or presence of IWP2. Scale bar = 50 μm. **(B):** Flow cytometry for CTNT (left) and real‐time polymerase chain reaction of NKX2.5 (right) comparing the dosage (treatment from day 1 to day 7) and time course (3 μg/ml treatment) of heparin treatment to promote cardiac differentiation in the absence of IWP2 treatment. Data are presented as the mean ± SD of three or more independent experiments. **(C):** Immunofluorescence (left) of CTNT (green) and NKX2.5 (red) as well as flow cytometry (right) with CTNT antibody, demonstrating that heparinase I blocked the effects of heparin and inhibited cardiomyocyte derivation. Data are presented as the mean ± SD of three or more independent experiments. Scale bar = 50 μm. **(D):** Heat map illustrating the promotion of cardiogenesis by heparin during differentiation at day 6 and day 10 in either absence or presence of IWP2. Abbreviations: CTNT, cardiac troponin T; ctrl, control; DAPI, 4′,6‐diamidino‐2‐phenylindole; hep, heparin; hpn, heparinase I.

To understand how heparin promotes cardiomyocyte differentiation, heparin was applied to hPSC differentiation at days 1–7 with or without Wnt inhibition (IWP2). Surprisingly, heparin treatment alone significantly increased the percentage of NKX2.5‐ and CTNT‐positive cells after 10 days of differentiation; however, IWP2 and heparin cotreatment produced the highest efficiency of cardiac derivation (Fig. 2A; supplemental online Video 2). The dosage screening suggested that the treatment of 3ug/ml of heparin from day 1–7 produced the most efficient cardiac derivation rate with a cell number yield comparable to IWP2 treatment (Fig. 2B; supplemental online Fig. 1D). The heparin used in the study (catalog no. H3149; Sigma‐Aldrich) is an unfractionated heparin sodium salt purified from porcine intestinal mucosa, which may possibly contain other contaminants 24. To confirm the specific effect of heparin in cardiac differentiation, we cotreated bacteroides heparinase I with heparin in differentiation media. Heparinase I is the enzyme that selectively cleaves the glycosidic linkage between hexosamines and uronic acids in heparin 25. The treatment with heparinase I dramatically blocked the effect of heparin in the promotion of cardiac differentiation (Fig. 2C). This result clarified that heparin, but not undefined contaminants, was the key factor to promote cardiac differentiation in treatment. To our surprise, the cardiac production under the IWP2 treatment was also significantly suppressed by heparinase I (Fig. 2C), which suggested that any endogenous heparin or heparan sulfate might also play a critical role in cardiac differentiation during the Wnt inhibition process. Furthermore, gene ontology analysis of microarray data from RNA samples on days 6 and 10 demonstrated that cardiogenesis was promoted by heparin with and without IWP treatment (Fig. 2D).

### Heparin Played Biphasic Roles in Modulating Wnt Signaling and Cell Growth During Cardiac Differentiation

**Figure 3:**
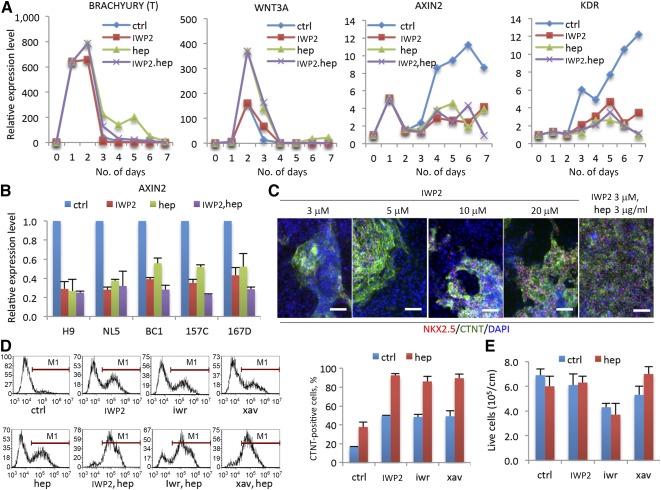
Heparin modulated canonical Wnt signaling during human pluripotent stem cell (hPSC) cardiac differentiation. **(A):** Heparin upregulated WNT3A expression at mesoderm induction stage (days 2–3) but inhibited canonical Wnt signaling downstream target AXIN2 and downregulated cardiovascular progenitor cell marker KDR during cardiac specification stage (days 3–6) as indicated by reverse transcription‐quantitative polymerase chain reaction (RT‐qPCR). Data are presented as the mean ± SD of three or more independent experiments. **(B):** RT‐qPCR for AXIN2 at day 5 with IWP2, heparin, and combination treatments in 5 hPSC lines. Data are presented as the mean ± SD of three or more independent experiments. **(C):** Immunofluorescence of NKX2.5 (red) and CTNT (green) at day 10 shows the differentiation efficiency among different dosages of IWP2 alone and in combination with heparin. Data are presented as the mean ± SD of three or more independent experiments. Scale bar = 50 μm. **(D):** Comparison of different Wnt inhibitors on cardiac differentiation with or without heparin treatment. Flow cytometry of CTNT‐positive cell population after 10 days of differentiation (left); percentage of CTNT positive cells is quantified (right). Data are presented as the mean ± SD of three or more independent experiments. **(E):** The yield of live cells with different Wnt inhibitor treatments after 10 days of differentiation in the absence (ctrl) or presence (hep) of heparin. Abbreviations: CTNT, cardiac troponin T; ctrl, control; DAPI, 4′,6‐diamidino‐2‐phenylindole; hep, heparin; iwr, IWR1; xav, XAV939.

To illustrate the function of heparin in mesoderm determination and cardiac specification, stage‐specific marker expression levels were measured by quantitative PCR (qPCR; Fig. 3A). The results showed that the expression of early mesoderm marker brachyury (T) peaked at days 1 and 2 but was not significantly affected when heparin was treated with IWP from day 1. However, heparin significantly upregulated Wnt3A expression at the mesoderm induction stage (days 2–3), whereas it inhibited Axin2, a downstream target of canonical Wnt signaling, at the later cardiac specification stage (days 4–6). This pattern indicated that heparin played biphasic roles in Wnt signaling regulation at different stages during cardiac differentiation, which is coincident to the endogenous change of Wnt signaling during mesoderm and cardiac differentiation. In addition, heparin also significantly downregulated, but did not totally block, the expression of cardiovascular progenitor marker KDR, which has been reported to be expressed at low levels in cardiac specific mesodermal cells 7, 26. All these gene expression patterns were reproducible in both human ESC line H1 and iPSC line ND2 (supplemental online Fig. 1E). The inhibition of Axin2 by heparin at day 5 was confirmed in 5 different human PSC lines (Fig. 3B). The inhibition of canonical Wnt signaling after day 3 made us speculate that higher dosages of IWP2 could produce enough cardiac differentiation efficiency without heparin treatment. However, increasing IWP2 dosage did not significantly enhance cardiac differentiation, as shown by CTNT and NKX2.5 staining (Fig. 3C), which suggested that inhibition of canonical Wnt signaling was not the only pathway affected by heparin.

We also evaluated the effects of multiple Wnt inhibitors on cardiac differentiation. Results showed that IWP2, IWR1, and XAV939 produced comparable efficiencies of cardiac differentiation in the presence of heparin, but treatment with IWR1 caused more cell death and yielded fewer cardiomyocytes (Fig. 3D, 3E; supplemental online Fig. 2A). Consistently, the expression of the cardiac progenitor marker, GATA4, was significantly upregulated by heparin at day 5, as shown in 7 different human PSC lines (supplemental online Fig. 1F). When we performed enriched function prediction by z‐score from microarray data comparing heparin treatment with the control, we found that heparin played biphasic roles in cell proliferation and differentiation between days 3 and 6. At day 3, heparin significantly increased cell growth but decreased cell death, whereas at day 6, heparin promoted cell differentiation but inhibited cell growth (supplemental online Fig. 3A). In the enriched function predictions, 577 genes were significantly up‐ or downregulated by heparin in either day 3 (*n* = 313 genes) or day 6 (*n* = 301 genes). Heat mapping indicated that 37 shared genes were regulated on both day 3 and day 6, whereas heparin had the opposite regulatory effect on most of these genes between days 3 and 6 (supplemental online Fig. 3B).

### Cardiomyocyte Differentiation on Defined Matrices From Multiple hPSC Lines

**Figure 4:**
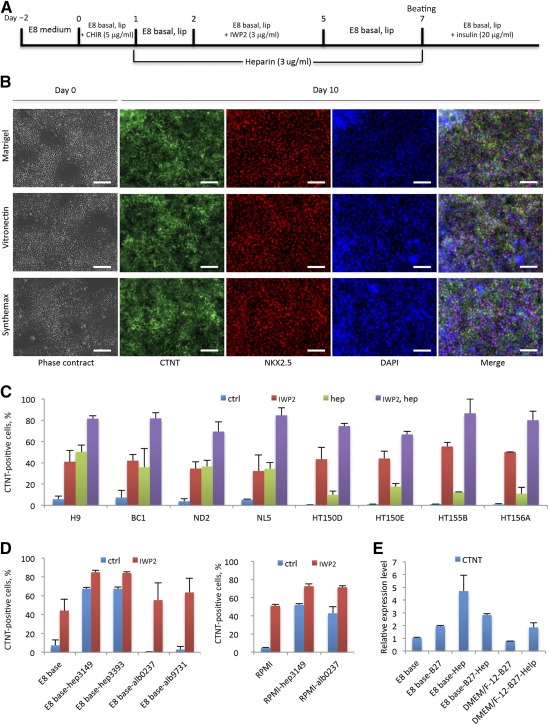
Heparin promoted cardiac differentiation from multiple human pluripotent stem cell (hPSC) lines in DMEM/F‐12‐based media. **(A):** Schematic of the hPSC differentiation protocol with modulation of Wnt signaling and treatment of heparin in chemically defined E8 medium. **(B):** Heparin promotes cardiomyocyte differentiation on Matrigel, vitronectin, and Synthemax matrices. Phase contrast images show the day 0 colony morphology before inducing differentiation. Immunofluorescence of CTNT‐positive (green) and NKX2.5‐positive (red) cardiomyocytes at day 10 of differentiation. Scale bar = 50 μm. **(C):** The cardiac derivation efficiency at day 10 as determined by flow cytometry with CTNT antibody. Data are presented as the mean ± SD of three or more independent experiments. **(D):** Flow cytometry using CTNT antibody comparing the effects of different batches of heparin and recombinant human albumin on cardiac differentiation with E8 basal medium (left) or RPMI medium (right) in either absence or presence of IWP2 treatment. The batch numbers of the reagents are listed. Data are presented as the mean ± SD of three or more independent experiments. **(E):** Quantitative reverse transcription‐polymerase chain reaction for CTNT comparing the effects of heparin and B27 (without insulin) on cardiomyocyte differentiation in E8 basal medium and DMEM/F‐12 medium. Data are presented as the mean ± SD of three or more independent experiments. Abbreviations: alb, albumin; B27, B27 without insulin; CTNI, cardiac troponin I; CTNT, cardiac troponin T; ctrl, control; hep, heparin; DAPI, 4′,6‐diamidino‐2‐phenylindole; DMEM, Dulbecco's modified Eagle's medium; lip, chemically defined lipid; RPMI, Roswell Park Memorial Institute medium.

According to these results, we proposed a working model of heparin's effect on cardiomyocyte differentiation in chemically defined conditions (supplemental online Fig. 4). In this model, heparin has a biphasic effect on regulating canonical Wnt signaling during different stages, and its Wnt inhibitory role significantly enhanced cardiac differentiation. Based on this model, we established an efficient cardiac differentiation method with a commercially available xeno‐free E8 medium platform (Fig. 4A; supplemental online Fig. 2B). In this method, no albumin was used, and the treatment of heparin dramatically enhanced cardiac derivation efficiency from 50% to as high as 95% troponin T‐positive cells after 7–10 days of differentiation (Fig. 1F).

Because of its widespread use, Matrigel was the main extracellular matrix material used in hPSC maintenance and differentiation in this study. However, Matrigel is undefined and still contains animal products. To develop a xeno‐free chemically defined protocol, we further tested the heparin‐based cardiac differentiation method on defined matrix surfaces, such as xeno‐free human recombinant vitronectin‐and Synthemax‐coated plates (Corning Life Sciences, Tewksbury, MA, https://www.corning.com) (Fig. 4B). The results indicated that our protocol worked comparably on undefined Matrigel and defined vitronectin and Synthemax.

Meanwhile, because this protocol was developed on the human H1 ESC line, we also tested H9 ESCs and more than 10 human patient iPSC lines (containing both male and female lines derived by multiple methods). The results confirmed that heparin significantly enhanced cardiomyocyte derivation efficiency with or without IWP2 treatment. With the combination of heparin and IWP2 treatment, all the tested hPSC lines had the capacity to generate 80% or greater troponin T‐positive cardiomyocytes after 10 days of differentiation (Fig. 4C; supplemental online Video 3).

After establishing heparin as an efficient cardiac‐promoting reagent, we revisited the role of B27 and albumin in cardiac differentiation. Most current protocols in the literature used Roswell Park Memorial Institute (RPMI) basal medium for cardiac differentiation, because DMEM/F‐12‐based medium was reported to be less efficient 8, 11. However, our study demonstrated that E8 basal medium (DMEM/F‐12 based) could yield high efficiency upon heparin treatment. When the effects of heparin and albumin in these two medium platforms were compared, we found that heparin promoted cardiac differentiation in both basal media with or without IWP2, whereas albumin's effect was base‐medium dependent. Albumin had a positive impact in RPMI medium with or without IWP2, whereas in E8 basal medium, albumin alone could not promote cardiac differentiation and had only a marginal improvement in the presence of IWP2 (Fig. 4D). This suggests that heparin and albumin could have different mechanisms of regulating cardiac differentiation, and albumin's effect, therefore, would be base medium dependent. In addition, we also demonstrated that in E8 or DMEM/F‐12‐based medium under treatment with IWP2, B27 (without insulin) was not as efficient as heparin in promoting hPSC cardiac differentiation (Fig. 4E). It was recently reported that albumin had an inhibitory effect on Wnt signaling and careful modulation of Wnt activation/inhibition alone allowed efficient cardiac differentiation for specific hPSC lines in the absence of albumin 13. However, such methods might need individualized optimization for different cell lines because different cells often respond to Wnt inhibition differently (Figs. 3B, 4C). Instead, we found that the presence of heparin along with Wnt inhibitor promoted efficient cardiac differentiation for all the lines that were tested with a simple formula (Fig. 4C), and such treatment could help establish a robust and uniform differentiation platform for different cell lines.

**Figure 5:**
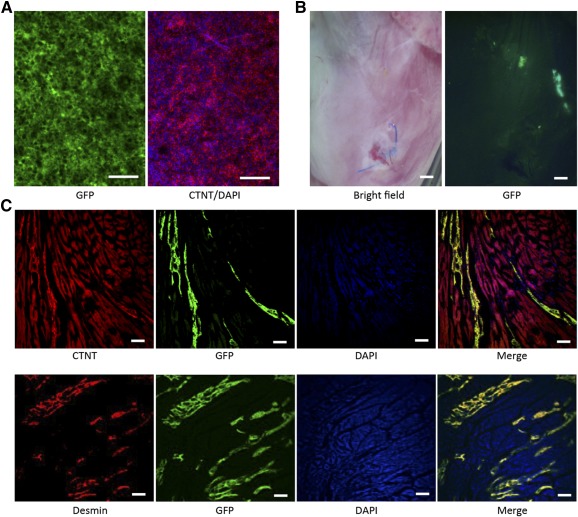
In vivo transplantation of human pluripotent stem cell (hPSC)‐derived cardiomyocytes in NSG mice. **(A):** GFP‐labeled human induced PSC (hiPSC)‐derived cardiomyocytes (green: GFP labeled hiPSCs; red: CTNT; blue: DAPI stain). Scale bar = 50 μm. **(B):** Light microscopy (left) and GFP fluorescence (right) of a NSG mouse heart 2 weeks after infarction and hiPSC‐derived cardiomyocyte injection. Scale bar = 0.4 mm. **(C):** Immunohistochemistry for CTNT (antibody recognizes both human and mouse antigens; top) and desmin (antibody recognizes only human, and not mouse, antigen; bottom) on cross sections from the infarcted area of a NSG mouse heart performed at 7 weeks after injection of enhanced GFP‐labeled induced pluripotent stem cells‐derived cardiomyocytes. Abbreviations: CTNT, cardiac troponin T; DAPI, 4′,6‐diamidino‐2‐phenylindole; GFP, green fluorescent protein.

To evaluate the potential application of clinical transplantation, we further tested the in vivo survival and incorporation of these iPSC‐derived cardiomyocytes in an animal model. Beating cardiomyocytes were derived from enhanced green fluorescent protein (EGFP)‐labeled iPSCs 27 and injected into the myocardium of immunodeficient NSG mice after ligation of the left anterior descending artery (Fig. 5A, 5B). At 2 or 7 weeks after transplantation, the mice were sacrificed and the cross‐sections of the hearts were stained with antibodies of CTNT and desmin. As shown in Figure 5C, EGFP‐positive cells still existed in the infarcted area and the fluorescence was overlapped with CTNT and desmin staining. The result indicated that the iPSC‐derived cardiomyocytes could survive and incorporate into infarcted mouse heart, which is promising for future cellular therapy applications.

## Discussion

In this study, we used an albumin‐free culture medium to identify heparin as a cardiac‐promoting reagent. As a proteoglycan that is broadly present in the whole body and serum, heparan sulfate molecules are expressed endogenously and are components of the cellular surface and extracellular matrix. Most biological functions of heparan sulfate are mediated by interactions with proteins that can be promoted or inhibited by exogenous heparin 20. One of the major functions of heparan sulfate proteoglycans is the ability to regulate the activity of growth factors by various mechanisms. They can act as a coreceptor with the growth factor cell surface receptor, controlling growth factor diffusion through the extracellular matrix, and extending growth factor half‐lives 18, 28, 29. Heparin has been reported to regulate neural development, cardiomyocyte hypertrophy, and heart function 30, 31, 32, 33, but it has never before been linked to cardiomyocyte cell fate determination, to our knowledge. With heparin, cardiomyocytes could be generated with chemical activation and inhibition of Wnt signaling in chemically defined culture media. This process could be carried out either on an undefined surface like Matrigel or on defined surfaces such as recombinant vitronectin and synthetic arginine‐glycine‐aspartic acid (RGD) peptides (Fig. 4B). It is important to note that like most unfractionated or low‐molecular‐weight heparins used in clinical practice and research, the heparins used in this study were also unfractionated and from porcine sources, which may cause concern about its purity and functional specificity. However, our experiments with the cotreatment of heparinase clarified the specific function of heparin in the promotion of cardiac differentiation. The data showing that different batches of heparins from Sigma‐Aldrich worked comparably also demonstrate the consistent effects of heparin in this process. Meanwhile, synthetic heparin is now potentially available 34, 35; it is conceivable that a completely chemically defined animal‐free production process could be developed with the synthetic heparin in the future.

Besides the practical value of heparin in cardiac differentiation, this study also revealed the biphasic effect of heparin on Wnt signaling as well as on cell proliferation and differentiation at different cardiac differentiation stages (Fig. 3A; supplemental online Fig. 3). Such effects were not previously reported in cell culture, although the interaction between heparin and Wnt signaling has been documented 28, 36, 37, and Wnt signaling was well known to play biphasic roles in heart development and cardiac development 8, 38, 39, 40. The new finding could help people appreciate the complexity of this cell culture platform because heparin is not only an important serum component but also a widely used reagent in cell differentiation and treatment of cell types such as mesenchymal, hematopoietic, and neural stem cells 41, 42, 43. Heparin's role in those studies was often attributed to the stimulation of FGF pathways, whereas the inhibitory effect of heparinase in differentiation with IWP treatment suggested that endogenous polyglycan‐dependent regulation plays an essential role in cardiac differentiation (Fig. 2C). Although the underlying molecular mechanisms are still under study, we are actively exploring heparin's alternative role in cell differentiation processes.

Our work demonstrated that albumin is not an essential factor for hPSC in vitro cardiomyocyte differentiation, and DMEM/F‐12‐based E8 basal medium could support efficient cardiac differentiation in the presence of heparin. The option to substitute albumin in cell culture is important for both basic and translational applications. Human recombinant albumin is expensive, and the quality could be quite variable from different batches and suppliers; animal components such as bovine serum albumins may also lead to an additional regulatory burden when developing clinically compliant material. Using heparin instead of albumin or B27 in cardiomyocyte derivation is beneficial in establishing an efficient and cost‐effective production process for future large‐scale requirements ($0.30 of heparin vs. $15 of human recombinant albumin or $50 of B27 minus insulin to make 500 ml of differentiation media). Several different lots of heparin were all confirmed to promote cardiac differentiation. This advance could eliminate inconsistencies due to source material batch differences that commonly occur in albumin‐ or B27‐related procedures and significantly reduce the cost of media.

## Conclusion

Our procedure provides an efficient and cost‐effective method to derive cardiomyocytes from hPSCs in fully chemically defined conditions. This could be a valuable tool for large‐scale production and future clinical applications.

## Author Contributions

Y.Lin: performance of cardiac differentiation, experiment design and screening for factors, immunostaining and flow cytometry analysis, data analysis, manuscript writing; K.L.L.: performance of cardiac differentiation, data analysis; B.M., K.J., and H.Y.: gene expression microarray performance and data analysis; M.K. and M.H.: performance of electrophysiology analysis; J.B.: immunostaining and flow cytometry analysis; W.X.: generation of GFP‐iPSC for differentiation and animal injection, immunostaining and flow cytometry analysis, performance of cardiac differentiation; Y.D.: performance of cardiac differentiation; C.L.: generation of GFP‐iPSC for differentiation and animal injection; Y.Lai: gene expression microarray performance and data analysis; J.Z.: generation of GFP‐iPSC for differentiation and animal injection, gene expression microarray performance and data analysis; M.R.: project conception; G.C.: project conception, performance of cardiac differentiation, experiment design and screening for factors, gene expression microarray performance and data analysis, manuscript writing.

## Disclosure of Potential Conflicts of Interest

The authors indicated no potential conflicts of interest.

## Supporting information

Supporting Information

## References

[1] Thomson JA , Itskovitz‐Eldor J , Shapiro SS et al. Embryonic stem cell lines derived from human blastocysts. Science 1998;282:1145–1147. 9804556 10.1126/science.282.5391.1145

[2] Takahashi K , Tanabe K , Ohnuki M et al. Induction of pluripotent stem cells from adult human fibroblasts by defined factors. Cell 2007;131:861–872. 18035408 10.1016/j.cell.2007.11.019

[3] Yu J , Vodyanik MA , Smuga‐Otto K et al. Induced pluripotent stem cell lines derived from human somatic cells. Science 2007;318:1917–1920. 18029452 10.1126/science.1151526

[4] Cherry AB , Daley GQ . Reprogrammed cells for disease modeling and regenerative medicine. Annu Rev Med 2013;64:277–290. 23327523 10.1146/annurev-med-050311-163324 PMC3629705

[5] Burridge PW , Keller G , Gold JD et al. Production of de novo cardiomyocytes: human pluripotent stem cell differentiation and direct reprogramming. Cell Stem Cell 2012;10:16–28. 22226352 10.1016/j.stem.2011.12.013 PMC3255078

[6] Liang P , Lan F , Lee AS et al. Drug screening using a library of human induced pluripotent stem cell‐derived cardiomyocytes reveals disease‐specific patterns of cardiotoxicity. Circulation 2013;127:1677–1691. 23519760 10.1161/CIRCULATIONAHA.113.001883 PMC3870148

[7] Yang L , Soonpaa MH , Adler ED et al. Human cardiovascular progenitor cells develop from a KDR+ embryonic‐stem‐cell‐derived population. Nature 2008;453:524–528. 18432194 10.1038/nature06894

[8] Lian X , Hsiao C , Wilson G et al. Robust cardiomyocyte differentiation from human pluripotent stem cells via temporal modulation of canonical Wnt signaling. Proc Natl Acad Sci USA 2012;109:E1848–E1857. 22645348 10.1073/pnas.1200250109 PMC3390875

[9] Dubois NC , Craft AM , Sharma P et al. SIRPA is a specific cell‐surface marker for isolating cardiomyocytes derived from human pluripotent stem cells. Nat Biotechnol 2011;29:1011–1018. 22020386 10.1038/nbt.2005 PMC4949030

[10] Ni TT , Rellinger EJ , Mukherjee A et al. Discovering small molecules that promote cardiomyocyte generation by modulating Wnt signaling. Chem Biol 2011;18:1658–1668. 22195568 10.1016/j.chembiol.2011.09.015 PMC3645312

[11] Burridge PW , Matsa E , Shukla P et al. Chemically defined generation of human cardiomyocytes. Nat Methods 2014;11:855–860. 24930130 10.1038/nmeth.2999 PMC4169698

[12] Laflamme MA , Chen KY , Naumova AV et al. Cardiomyocytes derived from human embryonic stem cells in pro‐survival factors enhance function of infarcted rat hearts. Nat Biotechnol 2007;25:1015–1024. 17721512 10.1038/nbt1327

[13] Lian X , Bao X , Zilberter M et al. Chemically defined, albumin‐free human cardiomyocyte generation. Nat Methods 2015;12:595–596. 26125590 10.1038/nmeth.3448 PMC4663075

[14] Chen G , Gulbranson DR , Hou Z et al. Chemically defined conditions for human iPSC derivation and culture. Nat Methods 2011;8:424–429. 21478862 10.1038/nmeth.1593 PMC3084903

[15] Francis GL . Albumin and mammalian cell culture: Implications for biotechnology applications. Cytotechnology 2010;62:1–16. 20373019 10.1007/s10616-010-9263-3 PMC2860567

[16] Beers J , Gulbranson DR , George N et al. Passaging and colony expansion of human pluripotent stem cells by enzyme‐free dissociation in chemically defined culture conditions. Nat Protoc 2012;7:2029–2040. 23099485 10.1038/nprot.2012.130 PMC3571618

[17] Tam PP , Loebel DA . Gene function in mouse embryogenesis: Get set for gastrulation. Nat Rev Genet 2007;8:368–381. 17387317 10.1038/nrg2084

[18] Rider CC . Heparin/heparan sulphate binding in the TGF‐beta cytokine superfamily. Biochem Soc Trans 2006;34:458–460. 16709187 10.1042/BST0340458

[19] Mikels AJ , Nusse R . Wnts as ligands: Processing, secretion and reception. Oncogene 2006;25:7461–7468. 17143290 10.1038/sj.onc.1210053

[20] Sarrazin S , Lamanna WC , Esko JD . Heparan sulfate proteoglycans. Cold Spring Harb Perspect Biol 2011;3:a004952. 21690215 10.1101/cshperspect.a004952 PMC3119907

[21] Spivak‐Kroizman T , Lemmon MA , Dikic I et al. Heparin‐induced oligomerization of FGF molecules is responsible for FGF receptor dimerization, activation, and cell proliferation. Cell 1994;79:1015–1024. 7528103 10.1016/0092-8674(94)90032-9

[22] Dabiri GA , Turnacioglu KK , Sanger JM et al. Myofibrillogenesis visualized in living embryonic cardiomyocytes. Proc Natl Acad Sci USA 1997;94:9493–9498. 9256510 10.1073/pnas.94.17.9493 PMC23235

[23] Yang X , Pabon L , Murry CE . Engineering adolescence: Maturation of human pluripotent stem cell‐derived cardiomyocytes. Circ Res 2014;114:511–523. 24481842 10.1161/CIRCRESAHA.114.300558 PMC3955370

[24] Rosania L . Heparin crisis 2008: A tipping point for increased FDA enforcement in the pharma sector?. Food Drug Law J 2010;65:489–501, ii [ii.] 24479237

[25] Linhardt RJ , Turnbull JE , Wang HM et al. Examination of the substrate specificity of heparin and heparan sulfate lyases. Biochemistry 1990;29:2611–2617. 2334685 10.1021/bi00462a026

[26] Kattman SJ , Witty AD , Gagliardi M et al. Stage‐specific optimization of activin/nodal and BMP signaling promotes cardiac differentiation of mouse and human pluripotent stem cell lines. Cell Stem Cell 2011;8:228–240. 21295278 10.1016/j.stem.2010.12.008

[27] Luo Y , Liu C , Cerbini T et al. Stable enhanced green fluorescent protein expression after differentiation and transplantation of reporter human induced pluripotent stem cells generated by AAVS1 transcription activator‐like effector nucleases. Stem Cells Translational Medicine 2014;3:821–835. 24833591 10.5966/sctm.2013-0212 PMC4073825

[28] Fuerer C , Habib SJ , Nusse R . A study on the interactions between heparan sulfate proteoglycans and Wnt proteins. Dev Dyn 2010;239:184–190. 19705435 10.1002/dvdy.22067 PMC2846786

[29] Kraushaar DC , Rai S , Condac E et al. Heparan sulfate facilitates FGF and BMP signaling to drive mesoderm differentiation of mouse embryonic stem cells. J Biol Chem 2012;287:22691–22700. 22556407 10.1074/jbc.M112.368241 PMC3391080

[30] Akimoto H , Ito H , Tanaka M et al. Heparin and heparan sulfate block angiotensin II‐induced hypertrophy in cultured neonatal rat cardiomyocytes. A possible role of intrinsic heparin‐like molecules in regulation of cardiomyocyte hypertrophy. Circulation 1996;93:810–816. 8641011 10.1161/01.cir.93.4.810

[31] Yoshioka J , Prince RN , Huang H et al. Cardiomyocyte hypertrophy and degradation of connexin43 through spatially restricted autocrine/paracrine heparin‐binding EGF. Proc Natl Acad Sci USA 2005;102:10622–10627. 16020536 10.1073/pnas.0501198102 PMC1180761

[32] Iwamoto R , Yamazaki S , Asakura M et al. Heparin‐binding EGF‐like growth factor and ErbB signaling is essential for heart function. Proc Natl Acad Sci USA 2003;100:3221–3226. 12621152 10.1073/pnas.0537588100 PMC152273

[33] Colombres M , Henríquez JP , Reig GF et al. Heparin activates Wnt signaling for neuronal morphogenesis. J Cell Physiol 2008;216:805–815. 18449906 10.1002/jcp.21465

[34] Linhardt RJ , Liu J . Synthetic heparin. Curr Opin Pharmacol 2012;12:217–219. 22325855 10.1016/j.coph.2011.12.002 PMC3496756

[35] Zulueta MM , Lin SY , Hu YP et al. Synthetic heparin and heparan sulfate oligosaccharides and their protein interactions. Curr Opin Chem Biol 2013;17:1023–1029. 24182748 10.1016/j.cbpa.2013.10.008

[36] Zhong X , Desilva T , Lin L et al. Regulation of secreted Frizzled‐related protein‐1 by heparin. J Biol Chem 2007;282:20523–20533. 17500071 10.1074/jbc.M609096200

[37] Ai X , Do AT , Lozynska O et al. QSulf1 remodels the 6‐O sulfation states of cell surface heparan sulfate proteoglycans to promote Wnt signaling. J Cell Biol 2003;162:341–351. 12860968 10.1083/jcb.200212083 PMC2172803

[38] Naito AT , Shiojima I , Akazawa H et al. Developmental stage‐specific biphasic roles of Wnt/beta‐catenin signaling in cardiomyogenesis and hematopoiesis. Proc Natl Acad Sci USA 2006;103:19812–19817. 17170140 10.1073/pnas.0605768103 PMC1750922

[39] Ueno S , Weidinger G , Osugi T et al. Biphasic role for Wnt/beta‐catenin signaling in cardiac specification in zebrafish and embryonic stem cells. Proc Natl Acad Sci USA 2007;104:9685–9690. 17522258 10.1073/pnas.0702859104 PMC1876428

[40] Willems E , Spiering S , Davidovics H et al. Small‐molecule inhibitors of the Wnt pathway potently promote cardiomyocytes from human embryonic stem cell‐derived mesoderm. Circ Res 2011;109:360–364. 21737789 10.1161/CIRCRESAHA.111.249540 PMC3327303

[41] Holley RJ , Pickford CE , Rushton G et al. Influencing hematopoietic differentiation of mouse embryonic stem cells using soluble heparin and heparan sulfate saccharides. J Biol Chem 2011;286:6241–6252. 21148566 10.1074/jbc.M110.178483 PMC3057799

[42] Stemple DL , Mahanthappa NK , Anderson DJ . Basic FGF induces neuronal differentiation, cell division, and NGF dependence in chromaffin cells: A sequence of events in sympathetic development. Neuron 1988;1:517–525. 3272178 10.1016/0896-6273(88)90182-1

[43] Benoit DS , Durney AR , Anseth KS . The effect of heparin‐functionalized PEG hydrogels on three‐dimensional human mesenchymal stem cell osteogenic differentiation. Biomaterials 2007;28:66–77. 16963119 10.1016/j.biomaterials.2006.08.033

